# *Entamoeba histolytica* under Oxidative Stress: What Countermeasure Mechanisms Are in Place?

**DOI:** 10.3390/cells6040044

**Published:** 2017-11-21

**Authors:** Erika Pineda, Doranda Perdomo

**Affiliations:** Laboratory of Fundamental Microbiology and Pathogenicity (MFP), University of Bordeaux, CNRS UMR-5234, 33000 Bordeaux, France; erika.pineda-ramirez@u-bordeaux.fr

**Keywords:** *Entamoeba histolytica*, nitric oxide, oxidative stress, energetic metabolism, endoplasmic reticulum

## Abstract

*Entamoeba histolytica* is the causative agent of human amoebiasis; it affects 50 million people worldwide and causes approximately 100,000 deaths per year. *Entamoeba histolytica* is an anaerobic parasite that is primarily found in the colon; however, for unknown reasons, it can become invasive, breaching the gut barrier and migrating toward the liver causing amoebic liver abscesses. During the invasive process, it must maintain intracellular hypoxia within the oxygenated human tissues and cellular homeostasis during the host immune defense attack when it is confronted with nitric oxide and reactive oxygen species. But how? This review will address the described and potential mechanisms available to counter the oxidative stress generated during invasion and the possible role that *E. histolytica*’s continuous endoplasmic reticulum (*Eh*-ER) plays during these events.

## 1. Introduction

*Entamoeba histolytica* is the causative agent of human amoebiasis. Infections by this parasite lead to distinct clinical manifestations, including diarrhea, dysentery, and hepatic liver abscess. Approximately 50 million people are affected by *E. histolytica* and approximately 100,000 die every year [1]. According to the 2013 Global Burden of Disease Study (GBD 2013), intestinal protozoan infections—cryptosporidiosis and amoebiasis—are the third leading cause of death worldwide [2,3]. These infections not only affect people from the developing world and tropical regions, but also first-world countries. People affected include high-risk groups, such as immigrants and travellers returning from countries of high endemicity (e.g., Central and South America, South-East Asia) [4,5] and indigenous people (i.e., Aboriginal Australians) [6]. The high incidence of infection in children from poorly sanitized areas of South Asia is a public heath problem. Recent reports established an incidence between 90–80%, and a reinfection higher than 50% in children from Bangladesh [7]. A similar result was observed in the North Eastern states of India, where the incidence of infection was 55%, and in this study it was shown that children under 15 years old are particularly at risk of infection [8]. This information highlights that amoebiasis is a considerable health burden that targets children under development with thus considerable long-term effects [9].

The infection process begins with the ingestion of fecally contaminated water or food, in which *E. histolytica* cysts can be found. During this first step in the human body, the cyst will start differentiating into trophozoites upon its passage through both the stomach and small intestine. Then, once in the colon, the trophozoites bind to enterocytes, colonize the colon, and survive. For unknown reasons, the invasion process into the tissues starts when the trophozoites residing in the colon disrupt the gut barrier. These events need parasite adhesion to human cells, which is carried out by a set of proteins called virulence factors that are present at the trophozoite surface. The Gal/GalNAc lectin mediates the mechanisms for both adherence and cytotoxicity toward host cells [10] and prevents amoeba elimination by activating the complement membrane attack complex [11]. Other adherence molecules, such as the lysine and glutamic acid rich protein 1 (KERP1), also contribute to the trophozoite adherence [12]. Close contact between the trophozoite and the host cell is necessary for amoebic cytolytic effectors to act. Cysteine proteases cleave the host’s colonic mucin, [13] leading to the disruption of the mucus layer defensive barrier and subsequently to the destruction of the colonic epithelium [14]. Cysteine peptidases weaken the host cell’s tight junctions [15] and inactivate immunoglobulins [16] and components of the complement cascade, thereby contributing to the evasion of the host’s immune response [17]. Furthermore, the parasite’s selective ability to nibble on live human cells [18] and phagocytose erythrocytes, bacteria, [19] and other surrounding cells [20,21] not only contributes to the invasive process but also to parasite survival and establishment in the host.

For research purposes, *E. histolytica* parasites were isolated from an asymptomatic carrier (i.e., the non virulent Rahman strain) and from a symptomatic patient (i.e., the virulent HM1:IMSS strain). Following the sequencing and annotation of the *E. histolytica* genome [22], a subset of comparisons between virulent and non virulent *E. histolytica* analyses has aided in the investigation of the gene expression profile of the parasite in the context of infection, thereby helping to understand the molecular basis of amoebiasis. Furthermore, an ex vivo human intestinal model [23] has been essential for analyzing the changes involved in colonic invasion [14]. The mechanisms by which the parasite can deliver virulence factors continuously to the cell surface, escape the host’s immune system, and survive its surrounding stress are not fully characterized. This review will address the mechanisms available to counter the oxidative stress generated during invasion and the possible role that *E. histolytica*’s endoplasmic reticulum (Eh-ER) plays during these events. 

## 2. Amoeba Survival

*Entamoeba histolytica* is an anaerobic parasite, which means that, once in the colon and during the invasion process, the trophozoite must maintain intracellular hypoxia in the human oxygenated tissues and maintain cellular homeostasis during the host immune defense attack when it is confronted with nitric oxide (NO) and reactive oxygen species (ROS). NO, which is produced from l-arginine by nitric oxide synthase (NOS), is released from activated macrophages; these immune cells can produce the cytotoxin that is responsible for killing *E. histolytica* [24]. Upon this attack from the host, the trophozoites stimulate cysteine synthase activity, inhibit glycolysis, and induce an ER-like stress response [25].

### Survival Mode on: What Metabolic Pathways are Affected and to What Purpose?

*E. histolytica* lacks glutathione [26], a major antioxidant molecule present in many organisms. Furthermore, the presence of trypanothione (conserved in kinetoplastids and present in Euglenids), an unusual form of glutathione that contains two molecules of glutathione joined by a spermidine linker, is being debated [27]. The major thiol present in *E. histolytica* is l-cysteine, and it mediates antioxidative defense [26]. Experiments have shown that, when amoebas are cultivated in the absence of l-cysteine, the intracellular ROS level is increased by more than three-fold [28], which highlights the relevance of this amino acid in the antioxidant defense against the host’s immune system. Additionally, l-cysteine protects the trophozoites from the oxidative shock produced by pharmacological treatment with metronidazole [29].

There are two ways for the parasite to obtain l-cysteine: through a de novo synthesis pathway or by the uptake from the extracellular milieu [30]. The de novo synthesis pathway involves two steps that are catalyzed by serine acetyltransferase (EC 2.3.1.30) and cysteine synthetase (EC 4.2.99.8). When the trophozoites are exposed to nitrosative stress, the biosynthetic pathway is capable of counteracting the increased amino acid demand by overexpressing cysteine synthetase [20]. Despite this mechanism, the contribution of the de novo synthesis pathway to intracellular cysteine concentration does not seem to be sufficient. If parasites are axenically cultivated in a medium lacking cysteine, the internal concentration of this amino acid is almost undetectable, suggesting that the trophozoite mostly depends on the external uptake from the media [28]. 

Besides its function as an antioxidant defense, cysteine plays a fundamental role in the conformation of iron–sulfur [Fe–S] clusters, which are cofactors for many enzymes in the parasite. These clusters are present in all organisms and are involved in many biological functions like electron transport, enzyme kinetics, nitrogen fixation, photosynthesis, and iron storage [31,32,33]. These clusters exist in different structural conformations, the simplest is an iron molecule bound to a protein by four cysteine atoms. The [Fe–S] clusters are vulnerable to oxidative and nitrosative stress [34]. It is well established that the [4Fe–4S]^2+^ centers, which are the most common clusters present in proteins, are oxidized to the form [3Fe–4S]^1+^, thereby releasing one Fe atom in this process. If the exposure to oxygen continues, the center irreversibly loses up to three Fe atoms, promoting the inactivation of the enzyme and affecting the biological pathway in which it is involved [35]. As many of the Fe–S clusters contain enzymes are involved in energy metabolism, its inactivation can lead to a decrease in adenosine triphosphate (ATP) production and viability. One example of this is what occurs during amoebic glycolysis. In *E. histolytica*, the energy metabolism is less complex than that in higher organisms because it does not have a mitochondria, a functional tricarboxylic acid cycle, or oxidative phosphorylation enzyme activities. *E. histolytica*’s main energy source is glucose; therefore, glycolysis is the principal pathway to obtain ATP [36]. For this process, *E. histolytica* Fe–S enzyme, pyruvate:ferredoxin oxidoreductase (PFOR, EC 1.2.7.1), catalyzes the oxidative decarboxylation of pyruvate to produce acetyl-CoA, transferring the electrons to two ferredoxin molecules, and this oxidized ferredoxin is involved in the activation of metronidazole [37]. This enzyme PFOR possesses three [4Fe–4S]^2+^ clusters that are inactivated by ROS and NO. The susceptibility of PFOR to ROS has been assessed in anaerobic microorganisms [38,39]. In example, the PFOR from *Desulfovibrio africanus* is not affected by ROS, which is due to the presence of an extra domain in the C-terminal region that overlays the Fe–S cluster region and protects against ROS exposure [39]. The amoebic PFOR is highly susceptible to O_2_, H_2_O_2_, and NO. In the conditions assayed, the inactivation of PFOR was reversible for ROS, but not for NO [40,41]. This can be due to the higher concentration assayed for the latest or because NO promotes a faster destabilization of the cluster.

**Figure 1:**
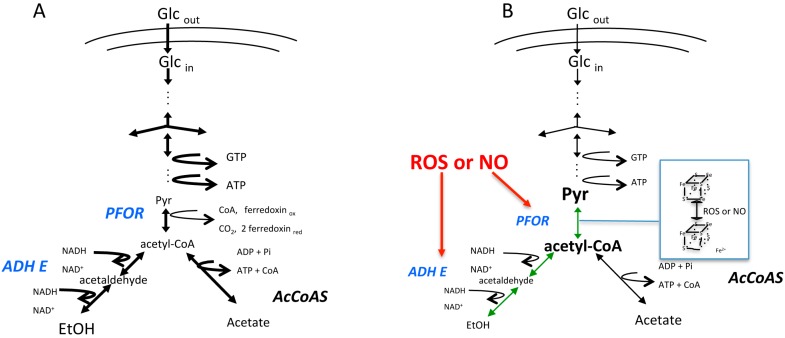
Schematic representation of the glycolytic pathway in *E. histolytica.* The diagram depicts the pathways that are present under (**A**) microaerophilic control conditions or (**B**) after exposure to reactive oxygen species (ROS) or nitric oxide (NO) (in red). The thickness of the arrows represents the relative flux rates through the enzymatic reactions. The size of the metabolite abbreviation represents its relative concentration within the trophozoites. Under stress condition, pyruvate:ferredoxin oxidoreductase (PFOR) and aldehyde/alcohol dehydrogenase (ADHE) (in blue) are inhibited. The metabolic intermediaries before PFOR and ADHE reactions (in green) are accumulated with the concomitant decrease in ethanol (EtOH) and adenosine triphosphate (ATP) production. Glc_out_, external glucose; Glc_in_, internal glucose. Adapted from [47].

The acetyl-CoA generated by PFOR is further reduced to ethanol by a bifunctional aldehyde/alcohol dehydrogenase (ADHE, EC 1.2.1.10) or converted to acetate by the ADP forming acetyl-CoA synthetase (AcCoAS, EC 6.2.13) [42] (Figure 1A). The structure of EhADHE is similar to the bifunctional ADHE present in *E. coli* [43], where the N-terminal domain contains aldehyde dehydrogenase activity, whereas the C-terminal domain possesses alcohol dehydrogenase activity and an Fe^2+^ binding site. The stability of both domains and the presence of one Fe^2+^ atom are necessary for catalysis, especially for aldehyde dehydrogenase activity [44]. This enzyme is also inhibited by ROS and NO (Figure 1B), as has also been demonstrated for the bacterial enzyme [45]. In contrast to what happens with amoebic PFOR, the inactivation of ADHE is irreversible and to reestablish this enzyme’s activity, a de novo synthesis mechanism might be needed [41] (Figure 1B). The inhibition of ADHE decreases the glycolytic flux and compromises both ATP production and cellular viability [46]. The contribution of PFOR and ADHE to glycolytic flux was determined using a metabolic control analysis [46,47], and, according to this analysis, EhADHE is one of the main controlling steps in the glycolytic pathway, whereby its inhibition endangers the ATP supply [46]. Furthermore, an increase in glycerol biosynthesis has been observed in trophozoites exposed to oxidative stress [48], but the function of this increase has not been established yet. The effects that ROS and NO have on other metabolic pathways have not been determined, but it is expected that they might change to counteract the stress, especially those pathways involved in NADPH synthesis. 

## 3. Under Oxidative Stress, to Be or Not to Be Virulent, Matters

*E. histolytica* can be non-virulent by residing in the intestinal lumen or virulent by invading the tissue and inducing tissue damage. The virulent trophozoites that can adhere to the liver endothelium and migrate to the parenchyma through the liver sinusoidal endothelial cells induce an inflammatory response and foci of abscesses, finally developing amebic liver abscess (ALA). Although the host deploys a massive inflammatory response against *E. histolytica*, the parasite manages to survive within this environment. The capacity of the parasite to fight against ROS and NO will not only determine its survival, but also its virulence (for more insight see review [49]). To study the process of invasion and survival in the liver, an animal model was developed using the hamster (ALAH) [50]. This infection in the hamster is necessary to maintain the virulence factors expressed in the virulent amoeba strain, thus after ALAH infection, the trophozoites are isolated from the hamster liver and used as virulent trophozoites for experimental purposes. Interestingly, if trophozoites are continuously kept growing in vitro in axenic culture, they lose their virulence, thus their capacity to generate ALAH; this strain is known as the non virulent strain. The comparison between virulent and non virulent regarding gene differential expression has been discussed in many publications [51,52]. More recently, it was described that the marks associated with virulence and intestinal invasion are lost in non virulent amoebae. Virulent parasites freshly isolated from ALAH and exposed to human colon explants are capable of rapidly changing their gene expression profile by increasing the expression of genes related to carbohydrate and glycosylated residue metabolisms. This change in environmental conditions from the liver to the colon showed that modulation of the amoebic transcriptome is vital for parasite adaptation to allow for survival, growth, and invasive behavior [53]. On the contrary, virulence-attenuated trophozoites have an up-regulation of genes involved in the activation of SUMO and a downregulation of tRNA synthetases, suggesting that, in these trophozoites, the increase in proteasome activities and the downregulation of the translational machinery may be involved in gene regulation [53]. Furthermore, in the case of oxidative stress, the non virulent trophozoites possess a lower antioxidant capacity, and, importantly, when they are exposed to oxygen, the glycolytic flux is decreased in an irreversible manner [40], suggesting a role between virulence and survival mechanisms under host immune attack.

## 4. *E. histolytica* Endomembrane Network is a Puzzle of Vesicles

Trafficking pathways between organelles are highly conserved between mammalian, plant, and yeast cells. The vesicular transport hypothesis states that the transfer of cargo molecules between organelles of the secretory pathway is mediated by shuttling transport vesicles. Protein synthesis from the endoplasmic reticulum (ER), *en route* to the cell surface, invariably passes through the Golgi apparatus and the trans–Golgi network (TGN), a passage that is marked with post-translational modifications in the form of lumenal N-linked glycosylation [54,55]. This active process is coupled with all the endocytic and exocytic pathways; as such, the vesicular components are a reflection of their origin and they can carry protein cargo to be released upon reaching their destination. Considering the up-regulation of genes relevant to endomembrane dynamics in *E. histolytica* after the NO stress response, a closer look to the parasites trafficking network is important. Therefore, a quantitative proteomics approach to characterize the endomembrane network of this parasite was taken. The analysis was carried out using crude proteins extracts from *E. histolytica* intracellular vesicles, which range from 50 to 200 nm in size. Protein fractions were prepared from three independent biological experiments and processed for proteomics analysis identification, and then the protein abundance within the samples was quantified using intensity-based absolute quantification (iBAQ). This study gave a robust identification of *E. histolytica*’s endomembrane network, as it identified 1531 proteins, corresponding roughly to 20% of *E. histolytica* proteome, and it included the identification of the principal components of the ER and TGN [56].

### 4.1. E. histolytica Endomembrane Network is Affected by Oxidative and Nitrosative Stresses

The first study on the effects of ROS identified a general up-regulation of genes relevant to stress responses (i.e., thiol-dependent peroxidase [Eh29], superoxide dismutase [SOD], cysteine proteinase 5 (EhCP-5), G protein, heat shock protein 70 (HSP70), and peptidylprolyl isomerase). Overall, the results suggested that *E. histolytica* has several protective mechanisms to deal with oxidative stress during invasion [57]. Using transcriptomics, the Singh lab analyzed the trophozoites differential gene expression using whole-genome DNA microarrays. They compared HM1:IMSS *E. histolytica* strain (virulent) with the Rahman strain (non virulent), and showed that, upon interaction with H_2_O_2_ or nitrosative stress, the virulent strain up-regulated genes encoding for heat shock proteins, ubiquitin-conjugating enzymes, protein kinases, and small GTPases [58]. The non virulent strain experienced a higher sensitivity to oxidative stress conditions, suggesting that such conditions could be the outcome of a decrease in virulence. A follow-up analysis included the characterization of two proteins whose transcripts were up-regulated—a putative phospholipid transporting P-type ATPase/flippase (EhPTPA) and a stress-induced adhesion factor (EhSIAF). Overexpression of each protein in *E. histolytica* trophozoites enhanced parasite survival in response to oxidative stress and markedly increased the level of the EhPTPA protein. However, a clear role for amoebic functions was not specified, mainly because the overexpression of both proteins decreased the trophozoite’s motility and, in the case of EhSIAF, it reduced the parasites capacity to destroy human cell monolayers [59], contrary to what was expected.

In separate studies, the effect of only nitrosative stress was assessed by prolonged exposure of *E. histolytica* to the NO donor sodium nitroprusside (SNP), sodium nitrite (NaNO_2_), and sodium nitrate (NaNO_3_), all three induced apoptosis in the parasite. Although each product induced a cytotoxic effect after a short exposure, amoebic functions such as erythrophagocytosis were unaffected, as was amoebic proteolytic activity [60], suggesting that the endomembrane network functions of endocytocis and exocytosis were functional under stress. Importantly, in addition to the role of NO in the host immune defense, NO can also regulate protein–protein interactions [61], protein stability [62], and autophagy [63], through the *S*-nitrosylation of cysteine residues. Using the advantages of proteomics, Ankri’s lab used a technique [64] based on resin-assisted capture (RAC) of protein *S*-nitrosothiols (SNO), which was then coupled to mass spectrometry to detect, enrich, and identify *S*-nitrosylated *E. histolytica* proteins. Over 124 proteins were identified, and relevantly, the heavy subunit of the surface glycoprotein Gal/GalNAc lectin was also found. The study highlighted that *S*-nitrosylation of the Gal/GalNAc impaired the ability of the parasite to adhere to a HeLa cell monolayer and the ability of the purified Gal/GalNAc to bind to agarose beads in vitro. The analysis also corroborated previous studies showing that, upon NO exposure, the proteins involved in energy metabolism were also *S*-nitrosylated [65]. Considering the lethal effect of a high-dose NO exposure on *E. histolytica*, a complementary study using micromolar concentrations of NO demonstrated that the parasite can develop resistance to cytotoxic concentrations of NO and even adapt to the stressor [66]. This acquired resistance could favor the establishment of the parasite within the large intestine, where there is a continuous bombardment of macrophages and NO. The NO-adapted trophozoites had a two-fold longer duplication rate (20 h instead of 10 h), but importantly, they had developed resistance to activated macrophages. This adaptation is similar to the increased survival rate of trophozoites overexpressing EhPTPA and EhSIAF after exposure to H_2_O_2_ [59]. Transcriptomic analyses of NO-adapted trophozoites revealed 332 genes that were differentially expressed, showing an up-regulation of endomembrane-related genes (e.g., membrane traffic and ER-related genes) as well as genes related to cytoskeleton proteins (e.g., actin, actinin, and coronin).

Motility is an essential function for the survival of *E. histolytica* [67,68] not only for displacement and phagocytosis of host cells, but also for the intracellular trafficking of virulence factors [69]. Another project correlating the transcriptomic data of NO-exposed parasites to the proteomic data of NO-adapted trophozoites [70] showed an up-regulation in the gene expression of cytoskeleton proteins that were previously identified by proteomics. Mammalian actin *S*-nitrosylation can decrease actin polymerization and rearrange its cytoskeleton. Mass spectrometry analysis of NO-adapted proteins showed that the *E. histolytica* actin residue Cys286 was *S*-nitrosylated, suggesting that it could indeed impair the cytoskeleton functions in *E. histolytica*. These NO-adapted trophozoites had reduced virulence functions (i.e., erythrophagocytosis, cytophatic activity, and motility), similar to when actin is inhibited by Cytochalasin D, suggesting that NO exposure not only affects the endomembrane network [25], but also the cytoskeleton functions [70] by increasing its gene expression profile and modifying the proteins. Interestingly, once the NO pressure is removed, the trophozoites regained the same levels of virulence functions as the control parasites [70], demonstrating once again the ability of the amoeba to quickly adapt to different environments.

### 4.2. ER, Where Are You?

The typical ER morphology is described as an organized structure that is sectioned into branching tubules and flattened sacks, which are interconnected and located between the nuclei and the Golgi apparatus. During the protein folding process, ROS is produced as a by-product, conferring a natural environment of oxidative stress [71]. Considering that the protein folding process is dependent on redox homeostasis, the oxidative stress can perturb folding mechanisms and enhance the production of misfolded proteins, eventually becoming toxic for the cell and inducing apoptosis [72]. To prevent this, the classic ER developed the unfolded protein response (UPR), in which by up-regulation of certain genes related to folding, expression of chaperones calnexin/calreticulin (CRT) can prevent misfolded proteins from exiting the ER [73].

**Figure 2:**
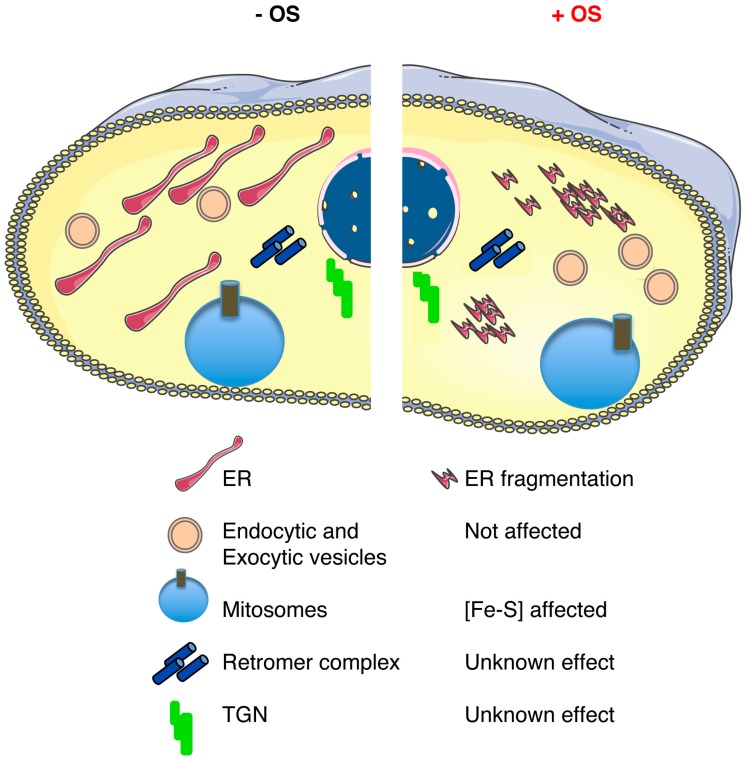
Schematic representation of the main components of the *E. histolytica* endomembrane network that is present in a trophozoite in the absence (−) or presence (+) of oxidative stress (OS). The diagram shows the described effects upon OS: ER fragmentation [22], a loss of adherence capacities of the Gal/GalNAc lectin [63], and a modification in the cytoskeleton proteins that could impair its function [64]. Notably, endocytic and exocytic activities remain functional under OS exposure. The effect of OS in the retromer complex, the TGN, and the structure of the mitosomes is unknown.

A particularity of *E. histolytica* is that the ER does not have a bonafide structural organization as just described; however, the ER-associated functions do exist in *E. histolytica*. Direct evidence for these functions were obtained from cellular biology experiments conducted to identify an ER-like structure in *E. histolytica*. Using a chimeric fluorescent protein expressed in trophozoites, a GFP fused with an N-terminal signal sequence from the Gal/GalNAc-specific *E. histolytica* adherence lectin followed by a FLAG epitope and a C-terminal ER retention peptide, KDEL (FLAG-GFP-KDEL), the authors showed, for the first time, that there was an ER-like structure in the parasite [74]. In a separate study, a homolog of another ER resident protein, CRT, was identified in *E. histolytica*. Further characterization of EhCRT, using a noncommercial antibody, also showed the existence of the ER in *E. histolytica* [75]. Expression and localization of both ER markers in the trophozoites—the chimeric FLAG-GFP-KDEL construct and the EhCRT—were found to be colocalized at a perinuclear intracellular compartment that appeared to be contiguous throughout the cytoplasm of the trophozoites, demonstrating that an ER-like organelle harboring both markers is present within vesicular compartments. Interestingly, under NO exposure to mimic the human macrophage response, the compartments labeled with FLAG-GFP-KDEL and *Eh*-CRT fragmented into small vesicles, as expected during ER stress, but importantly they remained localized together [25] (Figure 2). As mentioned above, one of the known responses to prevent oxidative stress in mammalian cells is the unfolded protein response pathway, more in detail, by the phosphorylation of the translation initiation factor eIF2α [76], which attenuates mRNA translation and prevents oxidative stress [77]. Considering that no classic ER structure has been described to date, studies regarding the unfolded protein response in *E. histolytica* have remained scarce. Part of the reason for this is that phylogenetic analyses show that none of the three classic unfolded protein responses (UPR) are present in *E. histolytica* [78]. In a recent study, identification and characterization of *E. histolytica* eIF2α (EheIF2α) indicated that part of the unfolded protein response could be present in this organism. The study showed that phosphorylation of *E. histolytica* eIF2α (EheIF2α) occurs in response to certain stress conditions, namely long-term serum starvation, long-term heat shock, and oxidative stress (exposure to H_2_O_2_). An increase in the level of phospho-EheIF2α (compared to control cells) was demonstrated only in trophozoites that experienced long-term serum starvation, long-term heat shock, or oxidative stress. Interestingly, researchers also compared between induced phosphorylation by serum starvation (stress condition) to no phosphorylation of Eh eIF2α by glucose deprivation (control condition). As expected, from studies in other organisms, under the stress condition, EheIF2α resulted in a significant reduction in dense polyribosomes and an increase in free ribosomes and monosomes, thus supporting the hypothesis that phosphorylation of EheIF2α attenuates translation. Unfortunately, the same experimental setup was not employed to determine if under oxidative stress conditions attenuation also occurs. The authors suggested that EheIF2α-based response system exists in *E. histolytica* and is activated in a stress-specific manner. Considering that *E. histolytica* have other unfolded protein response related genes that are homologs for eIF2β (EHI_153480) and eIF2γ (EHI_132880) and two presumptive eIF2α kinases (eIF2K) (EHI_109700, EHI_035950), it suggests that there could be an incomplete UPR pathway in this ancient parasite [79].

The machinery for the folding and production of secreted proteins is centralized in the ER, where nascent polypeptides can be transported from the ribosomes to and/or across the ER membrane. Sec61 comprises the main protein conducting channel, whereas signal-recognition particle receptor (SRPR), signal peptidases (SPC), the translocon-associated protein complex (TRAP), Sec63, and BiP comprise associated proteins that assist in signal sequence–mediated targeting, cotranslational translocation, and processing of nascent polypeptide chains. Exposing mammalian cells to NO shows that the ER folding machinery can be greatly affected [80], but, without a classic ER, it is difficult to evaluate the events that take place in the parasite. In *E. histolytica*, the translocation machinery is partly present. The Sec61α subunit was cloned [81], localized throughout the cytoplasm of the trophozoite with an occasional perinuclear localization [82] and also located at the plasma membrane of the parasite [83]. This localization is peculiar since the Sec61 α subunit localization has always been at the ER in other cells, but considering *E. histolytica* ER has a dispersed structural organization this is not a surprise. Interestingly, the downregulation of EhSec61 α was not lethal to the parasite, nor did it prevent trafficking of *Eh*CP-5. However, it did increase the levels of amoebapore at the plasma membrane [82], suggesting that protein secretion from the ER was not impaired, even though the protein is essential for ER translocation [84]. The *E. histolytica* proteomics analysis was able to identify several of the translocon machinery components, including SRPRα, SRPRβ, SPC2, SPC3, TRAPβ, Sec61α, Sec61γ, and BiP (Hsp70) [56], suggesting that, although some of the ER translocon proteins are present in *E. histolytica*, their function and localization might be different from what is described in mammalian cells or yeast.

## 5. Conclusions

It is well known that oxidative stress results from an imbalance in the antioxidant defense mechanisms and ROS levels. Although different organisms have developed pathways to restore cellular homeostasis, it is interesting to see how an anaerobic parasite, such as the amoeba, is able to fight against NO and ROS stress. Exposure to oxidative stress disrupts metabolic pathways, which compromises general metabolism, energy metabolism, protein folding, and endomembrane networks; however, their role and impact on other mechanisms of action has not been fully explored. Although each of the studies highlighted here show specific subset of genes that are differentially expressed with some common candidates, a step that is missing is to observe these changes using cellular biology. As such, it is relevant to point out that *E. histolytica* can fight against oxidative stress as a first response mechanism and it is even capable of adapting if slowly introduced to stress conditions, suggesting that much of what the expression profiles results show is that not only does the amoeba remains a parasite that will adapt according to the environmental pressures, but its virulence capacities adapt as well. These adaptations during the stress response could also hint at whether the trophozoite becomes invasive or undergoes encystation, as suggested by the levels of stress response proteins, hsp70 [85], and hsp90 [86] that fluctuate during stage conversion in *E. invadens*.

Notably, there is an active engagement of the *E. histolytica* cytoskeleton to provide backup for the amoebic survival functions (i.e., motility, erythrophagocytosis) and, more likely, although not yet explored, a relevant role in the endomembrane dynamics. Experiments have shown that there is an active engagement from the cytoskeleton and secretion pathway to deliver virulence factors to the plasma membrane, even under chemical stress conditions [69,87]. This process is contrary to what is detailed in mammalian cells, where the ER limits the folding and secretion when under stress to prevent ER overload. The question remains as to whether the trophozoite can also adapt its secretion pathway to maintain a constant delivery of virulence factors during oxidative stress responses.

To date, all of our input into amoebiasis has been in the context of a healthy human colon infection; however, development of amoebiasis in the context of inflammatory bowel disease, including ulcerative colitis and Crohn’s disease, is missing. Such inflammatory bowel disease scenarios favor a general inflammation in the gastrointestinal tract that can damage the intestinal barrier [88,89]. Under inflammatory bowel disease conditions, ER stress-induced unfolded protein response (i.e., activated ATF6) enhances an acute response in the liver, prolonging inflammation [86]. All of these responses could thus “help” the parasite migrate toward the tissues, developing ALA. Different infection environments could help us understand the mechanism of the adaptation and response from *E. histolytica* toward oxidative stress. Genetic or chemical intervention to reduce the capacity of *E. histolytica* to fight against oxidative stress is an avenue to explore more in detail as a way to treat or prevent the disease.

## Abbreviations

The following abbreviations were used in this manuscript:

AcCoAS	Acetyl-CoA synthetase
ADHE	Aldehyde/alcohol dehydrogenase
ALA	Amebic liver abscess
ATP	Adenosine triphosphate
CRT	Calreticulin
EhCP-5	Cysteine proteinase 5
ALAH	Hamster amebic liver abscess
NO	Nitric oxide
NOS	Nitric oxide synthase
OS	Oxidative Stress
SRPR	Particle receptor
SNO	Protein *S*-nitrosothiols
PFOR	Pyruvate:ferredoxin oxidoreductase
ROS	Reactive oxygen species
SPC	Signal peptidases
NaNO_3_	Sodium nitrate
NaNO_2_	Sodium nitrite
SNP	Sodium nitroprusside
SOD	Superoxide dismutase
Eh29	Thiol-dependent peroxidase
UPR	Unfolded Protein Response
